# Evolutionary trade-offs associated with copy number variations in resistance alleles in *Culex pipiens* mosquitoes

**DOI:** 10.1186/s13071-022-05599-8

**Published:** 2022-12-22

**Authors:** Pascal Milesi, Jean-Loup Claret, Sandra Unal, Mylène Weill, Pierrick Labbé

**Affiliations:** 1 Department of Ecology and Genetics, Evolutionary Biology Centre, Uppsala University, Norbyvägen, 18D, SE-752 36, Uppsala, Sweden; 2 Science for Life Laboratory (SciLifeLab), Uppsala, Sweden; 3 Institut Des Sciences de L’Évolution de Montpellier (UMR 5554, CNRS-UM-IRD- EPHE), Université de Montpellier, Cedex 05, Montpellier, France; 4 Institut Universitaire de France, 1 Rue Descartes Cedex 05, 75231 Paris, France

## Abstract

Organophosphate and carbamate insecticides have largely been used worldwide to control mosquito populations. As a response, the same amino acid substitution in the *ace-1* gene (G119S), conferring resistance to both insecticides, has been selected independently in many mosquito species. In *Anopheles gambiae*, it has recently been shown that the G119S mutation is actually part of homogeneous duplications that associate multiple resistance copies of the *ace-1* gene. In this study, we showed that duplications of resistance copies of the *ace-1* gene also exist in the *Culex pipiens* species complex. The number of copies is variable, and different numbers of copies are associated with different phenotypic trade-offs: we used a combination of bioassays and competition in population cages to show that having more resistance copies conferred higher resistance levels, but was also associated with higher selective disadvantage (or cost) in the absence of insecticide. These results further show the versatility of the genetic architecture of resistance to organophosphate and carbamate insecticides around the *ace-1* locus and its role in fine-tuned adaptation to insecticide treatment variations.

**Graphical Abstract:**

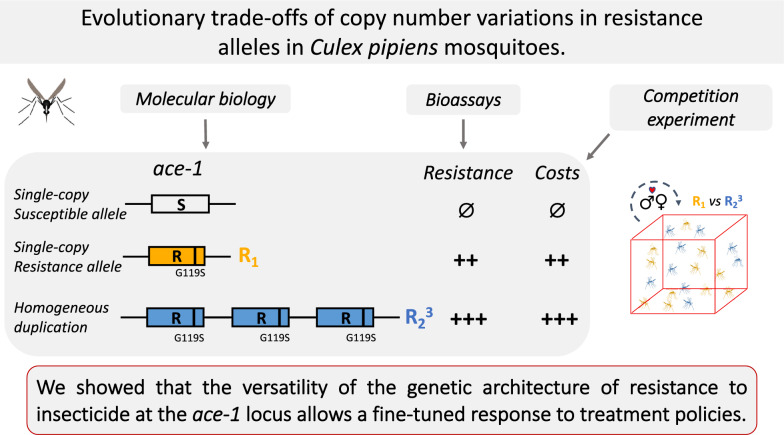

**Supplementary Information:**

The online version contains supplementary material available at 10.1186/s13071-022-05599-8.

## Background

Whether for sanitary or economic (agriculture, tourism) reasons, pest and vector species have been the target of intense xenobiotic exposure to control their populations. As a response, resistances have been selected for and have spread worldwide in many diverse organisms [1–3]. Resistance has been the focus of many studies with a management perspective, in particular in vector species where long-term monitoring and resistance evolution surveys took place, for instance in mosquitoes ([3] and references therein). However, resistance to insecticides also provides a wealth of information for evolutionary biologists as it is an iconic example of rapid adaptation to a new environment.

Mutations conferring resistance to insecticides are adaptive in the sense that they provide greater fitness in presence of insecticide. However, in mosquitoes, most of the mutations conferring resistance described so far are also disadvantageous in the insecticide-free environment [4–9]. For instance, the mutated allele could encode for a protein that is less efficient than the susceptible one, or metabolic equilibria could be dysregulated. Both can result in strong deleterious effects [3], affecting many different life history traits (hereafter termed ‘selective disadvantage;’ see useful criticism of use of the term ‘cost’ for these selective disadvantages by Lenormand et al. [10]). Because both advantages and disadvantages can vary between resistance mutations, they convey different evolutionary trade-offs in different environments (e.g. [11, 12], in particular regarding insecticide treatment intensities [5, 13]). For instance, intermediate treatment intensities would favor heterogeneous duplications over single-copy resistance alleles, despite the fact that they confer a lower resistance level, because they are also associated with a much lower fitness cost. However, heterogeneous duplications are outcompeted by single-copy resistance alleles when selective pressure is high because of a too low resistance level [5, 7]. In a constant environment, and as time passes, new alleles, with a more favorable evolutionary trade-off, and/or compensatory mutations are expected to be selected for (e.g. [14] in *Lucilia cuprina*, [11, 15–17] in *Culex pipiens* species complex and [18] in *Anopheles gambiae*). These contemporary evolutions of insecticide resistance are thus iconic examples of adaptive trajectories (“adaptive walk” in Orr [19]). The resistance alleles selected along these adaptive walks can result from simple nucleotide substitutions (e.g. affecting the target of insecticides), but various genetic architectures can also be selected for, for instance homogeneous duplications (aka gene amplification) or heterogeneous duplications [3, 20]. Different genetic architectures of resistance can be selected for precisely because they are associated with different evolutionary trade-offs [5, 21, 22]. Finally, the variation of treatment intensities in time and/or space can also generate balancing selection patterns that could (i) allow for the maintenance of susceptible and resistance alleles polymorphism in natural populations, (ii) select for alleles with more generalist trade-offs (e.g. [9, 10, 16]), but also (iii) maintain resistance allele polymorphism [23].

Among the most commonly used families of insecticides worldwide, organophosphate (OPs) and carbamate (CXs) insecticides target acetylcholinesterase (AChE1), encoded by the *ace-1* locus, which terminates cholinergic neurotransmission by hydrolysis of acetylcholine (ACh). These insecticides bind to AChE1, thereby impeding ACh degradation and inducing death by tetany. Point mutations modifying the conformation of the AChE1 active site and preventing the binding of the insecticide have been selected for in many vector and pest species. In particular, the same amino acid substitution (G119S) has been repetitively and independently selected in many mosquito species [24, 25]. While conferring resistance to OPs and CXs, the G119S mutation has also been shown to decrease the activity of the resistant acetylcholinesterase (AChE1R) by about 60% compared with the wild-type protein (AChE1S) in both the West Nile virus vector *Culex pipiens* sensu lato and the malaria vector *An. gambiae* [26, 27]. In both species, this drastic reduction in affinity with its natural subtract probably explains a large part of the selective disadvantage endured by resistant mosquitoes in absence of insecticides compared to susceptible ones [4, 7, 12, 28, 29].

In recent studies of *An. gambiae*, all alleles carrying the G119S mutation (R alleles) have been found to be part of homogeneous duplications (several resistance copies in tandem, R^x^ alleles) or of heterogeneous duplications (pairing a susceptible and a resistance copy, D alleles), i.e. they were never found in the natural population in a single-copy state [18, 22, 30].

In the *C. pipiens* species complex, two different R alleles have been found widely spread across natural populations [23]: R_1_ is found in *C. pipiens* sensu stricto all over Europe and the Mediterranean area, and R_2_ is found worldwide in *Culex quinquefasciatus*. They are also found in many D alleles associated with local susceptible variants [23]. However, the possibility that some R alleles could, as in *An. gambiae*, actually be part of homogeneous duplications, as well as what phenotypic effects these different genomic architectures could induce, has not yet been investigated in *C. pipiens* species complex.

In the present study, we isolated the R_1_ and R_2_ alleles in laboratory strains sharing the genetic background of the susceptible reference strain, SLAB. We first showed that, while R_1_ is found in a single-copy state, R_2_ is part of a homogeneous duplication carrying three R copies. We then investigated the phenotypes conferred by these different alleles (protein activity, resistance level and dynamics in absence of insecticides) and showed that different evolutionary trade-offs are associated with the different genomic architectures. We finally discuss the implication of the present study from both evolutionary biology and more applied perspectives.

## Materials and methods

### Mosquito strains

Three mosquito laboratory strains were used in this study: SLAB [31], SR [32] and SRQ (this study). SLAB is fixed for a single-copy susceptible allele (S_SLAB_, isolated in California, *C. quinquefasciatus*). SR is fixed for R_1_ [24], a resistance allele isolated from Southern France and found in *C. pipiens* s.s. all over Europe and around the Mediterranean Sea [23, 33]. SRQ is fixed for R_2_ [24], a resistance allele found worldwide in *C. quinquefasciatus* [23] and isolated from a population from Martinique Island. The two resistance alleles were introgressed into the genetic background of the SLAB strain through at least 15 rounds of back-crossing. All strains thus share the same genetic background (> 99%) and differ from each other almost only in their *ace-1* locus (although recombination around the *ace-1* gene is not complete, most of the background effects would be eliminated).

All strains were regularly checked for contamination: DNA was extracted from pools of first-instar larvae (~ 200 individuals per pool) and molecular tests, specific for each *ace-1* allele (detailed below), were used to check the homogeneity of each strain.

### Genotyping

The various strains can be easily distinguished using a single PCR and different restriction fragment length polymorphisms (RFLPs). After DNA extraction, following the protocol in [34], a ~ 600-bp fragment of the *ace-1* gene, including intron 2 and most of exon 3 (with the resistance G119S mutation), was amplified using two generalist primers, Intron2dir1 and CpEx3rev, according to [35].

#### Susceptible vs. resistant

The G119S mutation creates an *AluI* restriction site [33] so that three genotypes can be distinguished (*AluI* RFLP test): susceptible homozygote (SS; one fragment, 597 bp), resistant homozygote (RR; two fragments, 496 and 101 bp) and heterozygote (RS; three fragments, 597, 496 and 101 bp); 5 µl of the PCR product was incubated for 2 h at 37 °C.

#### R_1_ vs. R_2_

The two different resistance alleles can be further distinguished by taking advantage of another single-nucleotide polymorphism (SNP) between R_1_ and R_2_ creating a *Bfa1* restriction site in R_2_ (Additional File 1). This second RFLP test (*Bfa1* RFLP test) distinguishes three genotypes, the homozygotes R_1_R_1_ (one fragment, 597 bp) and R_2_R_2_ (three fragments, 73, 132 and 392 bp) and the heterozygotes R_1_R_2_ (four fragments, 597, 73, 132 and 392 bp); 5 µl of the PCR product was incubated for 2 h at 37 °C.

### Gene copy number quantification

*ace-1* gene copy number was estimated for ten individuals of each resistant strain using quantitative real-time PCR (qRT-PCR). Two individuals from the SLAB-susceptible strain were also used as controls. After DNA extraction, we dispensed 250 ng of genomic DNA and 1.5 μl of reaction mixture containing specific primers, each at a concentration of 0.8 μM and 0.75 μl of Master Mix (LightCycler 480 SYBR Green I Master, Roche), into the wells of a 384-well plate with a Labcyte Echo525 dispenser. We performed qPCR as follows: activation at 95 °C for 8 min followed by 45 cycles of 95 °C for 4 s, 67 °C for 13 s and 72 °C for 19 s. Melting curves were generated by a post-amplification melting step between 70 °C and 95 °C for Tm analysis. All quantifications were replicated three times for each DNA template. Two loci were amplified for each individual: *ace-1* (primers: ‘Culexace1univdir3’ AGA AGG TGG ACG CAT GGA TG; ‘Culexace1univrev3’ ATC TGG ACG CAG GAG TTG G) and *ace-2*, a locus known to be in a single copy in these species (primers: ‘acequantidir’ GCA GCA CCA GTC CAA GG; ‘acequantirev’ CTT CAC GGC CGT TCA AGT AG) [36]. *ace-1* over *ace-2* copy-number ratios were determined by the advanced quantification method (LightCycler 480 software v.1.5.0). Standard reference curves were constructed with tenfold dilutions of a PCR product previously amplified with specific primers for each locus from SLAB DNA.

### Phenotyping

#### Protein activity

We measured acetylcholinesterase (AChE1) activity for 48 individuals of each resistance strain, using spectrophotometry [37]. Adult mosquitoes were decapitated, and each head was individually homogenized in 400 μl of a phosphate buffer (0.25 M, pH7) supplemented with 1% Triton X-100. Homogenates were centrifuged (9.3 g for 3 min), and 100 μl of the supernatant was dispensed into each of two wells of a 96-well microtitration plate. We added 10 μl of propoxur, a carbamate insecticide, at 10^−3^ M and 10^−1^ M (diluted in ethanol) into the first and second well, respectively. The plate was incubated for 15 min at room temperature. We then added 100 μl of substrate solution (25 mM sodium phosphate, pH 7.0, 0.2 mM DTNB, 0.35 mM sodium bicarbonate, 2.5 mM acetylthiocholine) to each well. AChE1 activity was estimated by measuring the change in optical density following the cleavage of acetylthiocholine, as described by [38]. Optical density at 412 nm was recorded every minute for 15 min with an EL 800 microplate reader (Bio-Tek Instruments, Inc.). The mean slope of each reaction was calculated with KCjunior v1.41.4 analysis software (Bio-Tek Instruments, Inc.) and was used as a measurement of AChE1 activity in each well. Individual AChE1 activity was computed as the average activity between the two wells. To avoid any block or sex confounding effects, individuals from both sexes and the two strains were evenly distributed in the plates.

#### Resistance level and bioassays

We used bioassays to assess the three strains’ resistance to an OP insecticide, temephos (PESTANAL^®^,96% purity). We incubated 20 late third-instar larvae for 24 h at 27 °C ± 2 °C in plastic cups containing 99 ml of distilled water to which we added 1 ml of insecticide solution at the required concentration (1 ml of ethanol in controls). Four replicates were performed for each concentration (from 0 to 0.07 g$\upmu$.ml^−1^ see Additional File 2 for the complete dataset). Larval mortality was recorded after 24 h of exposure. We used the *BioRssay* R package (v.1.0.0 [39], https://CRAN.R-project.org/package=BioRssay) to analyze the dose-mortality responses of the different *ace-1* alleles and calculate the LD_50_ of the different strains, i.e. the lethal dose for 50% of the sample.

#### Experimental evolution in population cages

Population cages were used to set up a competition experiment between the two resistance alleles in absence of insecticides. R_1_R_1_ and R_2_R_2_ individuals were crossed, and the resulting F1 (100% R_1_R_2_ individuals) was reared until adulthood under standard conditions (25 °C, > 60% humidity, 12:12 h light:dark). Adults were released into a new cage to mate freely and reproduce. Their offspring were raised and released in new cages to ensure discrete generations. The process was repeated 11 times (i.e. 11 generations) with three independent cages (i.e. replicates). Almost each generation, and for each cage, about a hundred second-instar larvae were genotyped using the *Bfa1* RFLP test (see above) to measure the frequency of each genotype (R_1_R_1_, R_1_R_2_, R_2_R_2_). Allelic frequencies were then computed from genotypic frequencies.

We estimated the relative fitness of the various genotypes (R_1_R_1_, R_1_R_2_ and R_2_R_2_) using a deterministic genetic model (reproduction and selection, 11 cycles, no drift). The model was adjusted to the data and optimized using a maximum-likelihood approach as in Milesi et al. [5, 23]. For the reproduction step, the frequency of each genotype in the larvae of generation *i* was computed from the allelic frequencies (*p*) in the gametes of the previous generation, assuming panmixia (Eq. 1):

1 $$\begin{array}{l} f ( {R_{1} R_{1} }) = p_{1}^{ 2} \\ f ( {R_{1} R_{2} } ) = 2 \times p_{1} \times p_{2} \\ f ( {R_{2} R_{2} } ) = p_{2}^{ 2}\end{array}$$

For each genotype *g* selection was then computed between larval and adult stages of generation *i* using the following genotype fitness: *w*_R1R1_ = 1, *w*_R1R2_ = 1 + *h.s* and *w*_R2R2_ = 1 + *s*, with *h* the dominance coefficient and *s* the selection coefficient, both varying between − 1 and 1 (Eq. 2):

2 $$f^{\prime}_{gi} = \frac{{f_{gi} \times W_{g} }}{{\sum (f_{gi} \times W_{g} )}}$$

The genotypic frequencies after selection were used to calculate the allelic frequencies in the gametes produced by the surviving adults (Eq. 3).

3 $$\begin{array}{l} {p}_{1}^{^{\prime}}=f\left({R}_{1}{R}_{1}\right)+\frac{f\left({R}_{1}{R}_{2}\right)}{2} \\ {p}_{2}^{^{\prime}}=(1-{p}_{1}^{^{\prime}})\end{array}$$

The first run of 100,000 simulations was used to explore the parameter space and provide the likelihood profile associated with different random pairs of *h* and *s* values (Eq. 4):

4 $$L = \mathop \sum \limits_{g} \mathop \sum \limits_{i} \left( {n_{gi} \times \ln \left( {f_{gi} } \right)} \right)$$

where *n* is the number of individuals of genotype *g* observed in the cages at generation *i*, and *f* is the frequency of the genotype *g* at generation *i* calculated for a given pair of *h* and *s* values using our deterministic genetic model. One million additional simulations were run with parameter ranges more limited around the maximal likelihood *h* and *s* pair to precisely estimate the coefficients and their support limits (rough equivalents to 95% confidence intervals), defined as *h* and *s* maximal and minimal values, resulting in a likelihood equal to the maximum likelihood minus 1.96, as in [5].

### Statistical analyses

All the statistical analyses were conducted using the R software (R Core Team, https://www.r-project.org/):

We used the following linear model to compare *ace-1* copy number between the various strains:

$${\gamma }_{ij}= \mu +{\alpha }_{j}+{\varepsilon }_{ij }\quad (\mathrm{mod}. 1)$$

with ${\gamma }_{ij}$ the number of copies of the *ace-1* gene in replicate *i* from strain *j*, $\mu$ the population mean, $\alpha$ is the fixed effect of strain *j* (SLAB, SR or SRQ) and ${\varepsilon }_{ij}$ the error term following a normal distribution $\mathcal{N}(0, 1)$.

We used the following linear model to test the significance of the difference in AChE1 activity between the SR and SRQ strains:

$${\gamma }_{ijkl}= \mu +{\alpha }_{j}+{\beta }_{k}+{\delta }_{l}+{\varepsilon }_{ijkl} \quad (\mathrm{mod}. 2)$$

with ${\gamma }_{ijkl}$ the AChE1 activity for individual *i* of strain *j* and sex *k* measured in plate *l*, $\mu$ the population mean and $\alpha$ the fixed effect of strain *j* (SR or SRQ). $\beta$ and $\delta$ are control for the fixed effects of sex *k* and plate *l*, respectively, and ${\varepsilon }_{ijkl}$ is the error term following a normal distribution $\mathcal{N}(0, 1)$.

For both models, the significance of the various terms was tested using likelihood ratio tests (LRTs) comparing the full model with a model without the tested effect (*‘anova’* function, R [40]). For both models, we also confirmed the absence of significant heteroskedasticity (‘*bptest’* function, *‘lmtest’* R package [41]) and that the models’ residuals followed a normal distribution (‘*shapiro.test*’ function, *‘stats’* R package).

Finally, we used binomial proportion tests (‘*prop.test*’ function, ‘*stats*’ R package) to assess whether the allele frequencies at the end of the experimental evolution in cages (i.e. after 11 generations) differed from initial frequencies of 0.5 (100% R_1_R_2_ individuals).

## Results and discussion

The goal of the present study was to investigate the potential existence of homogeneous duplications of the *ace-1* locus in the *C. pipiens* species complex, similar to those found in *An. gambiae*, to assess the phenotypic effects of different genetic architectures and their role in adaptation to insecticides.

### Higher ace-1 copy number partly restores protein activity levels

**Fig. 1:**
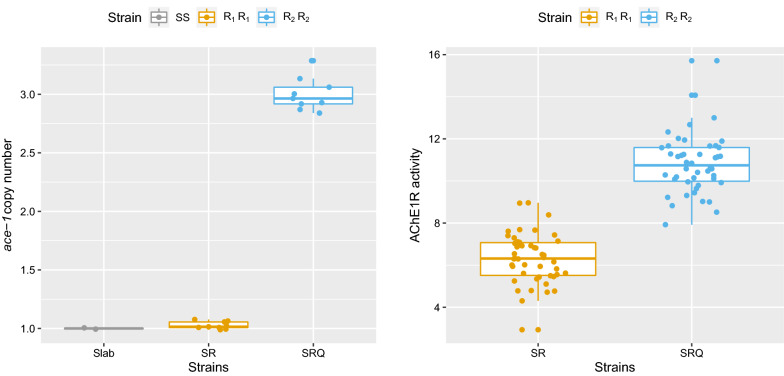
*ace-1* copy number and resistant acetylcholinesterase activity. Left panel is the number of copies of *ace-1* gene estimated using quantitative PCR for the susceptible strain, SLAB and the resistance strains SR and SRQ. Right panel shows the activity of the resistant acetylcholinesterase (AChE1R) estimated through spectrophotometry for the resistant strains SR (R_1_ allele, orange) and SRQ (R_2_^3^ allele, blue), respectively

We first quantified the number of *ace-1* copies in the three different strains, with the susceptible reference strain SLAB as a control. SLAB was found carrying a single-copy allele (mean = 1 ± 0.007 SD), and this was also the case for the resistant strain SR, carrying the R_1_ allele (mean = 1.03 ± 0.03 SD; mod. 1, *t* = 0.34, *p* = 0.74). However, we detected three copies of the *ace-1* locus in the SRQ strain (mean = 3 ± 0.14 SD), indicating that the R_2_ allele is a homogeneous duplication (R^x^), i.e. R_2_^3^ allele (mod. 1, *t* = 25.8, *p* < 0.001, Fig. 1A). While several R^x^ alleles (aka homogeneous duplications) have recently been described in *An. gambiae* populations, with two to nine *ace-1* copies [22, 30], this study is the first to report homogeneous duplications of the *ace-1* resistance allele in mosquitoes from the *C. pipiens* species complex.

We then investigated whether having more *ace-1* resistance copies would lead to higher activity of the resistant acetylcholinesterase (AChE1R, encoded by the R alleles). The activity A_R_ of the resistant acetylcholinesterase was significantly higher in the SRQ strain (A_R2_ = 10.8 ± 1.4_,_ three copies) than in the SR strain (A_R1_ = 6.3 ± 1.2; mod. 2, LRT: *F* = 269, *df* = 1, *p* < 0.001, Fig. 1B, Additional file 3: Table S1).

As in *An. gambiae* [22], it thus clearly appears that having a higher resistance copy number does increase the AChE1 protein activity. However, the protein activity did not increase in a strictly additive way with the number of copies of *ace-1*; R_2_^3^ activity is 1.67 times higher than that of R_1_, not three times as was expected. In previous studies in *An. gambiae* and *C. pipiens* species complex conducted on heterogeneous duplications, D, A_R_ was indeed found roughly proportional to the number of R copies [7, 21]. Here, the R_1_ and R_2_^3^ alleles differ not only by their copy number but also in their *ace-1* sequences (Additional file 1). As all R_2_^3^ copies are identical (no variation over 3 kb of the *ace-1* sequence in [23]), the departure from additivity observed could thus be explained by a lower per-copy activity for the proteins encoded by the *C. quinquefasciatus* R_2_^3^ allele compared to the protein encoded by the *C. pipiens* s.s. R_1_ allele. Alternatively, the expression of the *ace-1* gene in the SRQ strain could be somehow regulated. Finding more variation in copy number for the R_2_ allele, if any, would help settle this issue. For instance, in *An. gambiae*, for the homogeneous duplication R^x^, the relation between the number of resistance copies and ACHE1R activity is not strictly additive, even though all copies are identical [22], strongly suggesting that *ace-1* resistance copy expression is further regulated. Both hypotheses are not exclusive, and further studies are required to identify if and when regulation happens.

### Alternative genetic architectures confer different evolutionary trade-offs

**Fig. 2:**
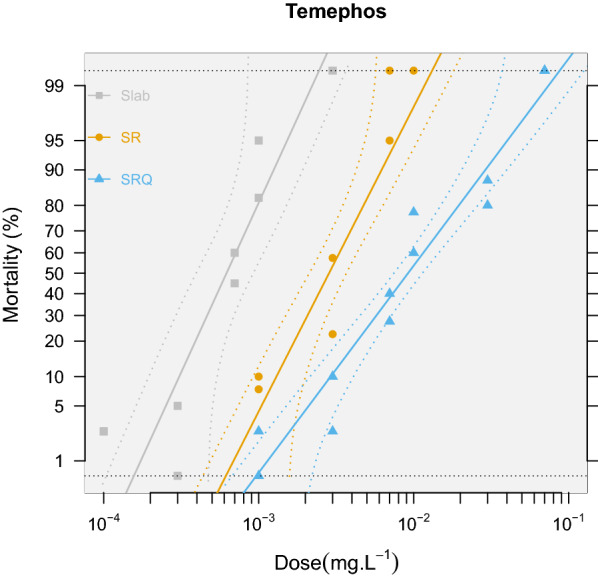
Bioassay analysis. Probit-transformed mortality rates as a function of the log-dose of insecticide [temephos (R), an OP insecticide]. Colors and point shapes indicate the different strains. The fit from the linear model is provided (solid line) along with the 95% confidence intervals (dotted lines). The linearity of the log-responses has been checked using *test.validity* = *T* in the “*mort.plot*” function from the “*BioRssay*” R package

We then compared the phenotypic consequences of copy number variation at the *ace-1* locus and first quantified the resistance levels associated with the different genotypes. As expected, both R_1_ and R_2_^3^ alleles confer insecticide resistance compared with the susceptible strain (RR_50_ = 4.6 [3.7–5.7], 95% CI and 15 [12–18], respectively), but more importantly, the duplicated allele conferred a significantly higher resistance level than the single copy allele (R_1_/R_2_^3^ resistance ratio RR_50_ = 3.2 [2.5–4.2], *X*_*2*_ = 61, *df* = 1, *p* < 0.001; Fig. 2 and Additional file 3: Table S2). We then addressed the selective disadvantages associated with the resistance alleles. Despite its obvious advantage in presence of insecticides, the SR strain was indeed repeatedly shown in previous studies to incur a strong selective disadvantage compared to SLAB in their absence (e.g. [28]). Rather than comparing both resistant strains to the susceptible one, we thus chose to directly assess whether R_2_^3^ incurred a stronger or lesser disadvantage than R_1_ in absence of insecticide through a competition experiment in population cages: this set-up allows an integrative assessment of their relative fitness over the full life cycle and ensures that genetic background effects associated to each strain (e.g. resulting from their fixation process) are strongly reduced, as the alleles are mixed in the individuals of each generation [5].

**Fig. 3:**
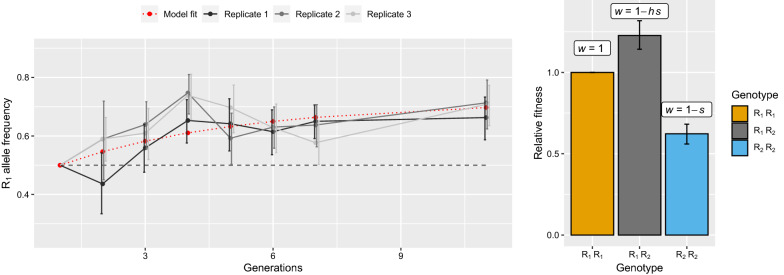
Dynamics of the resistance alleles and selection coefficients estimations. Left panel represents the dynamics of the R_1_ allele (SR strain) frequency (± SD) over generations, in competition with the R_2_ allele (SRQ strain) in absence of insecticide (the dots of the 3 replicates are slightly offset for easier reading). The R_1_ allele dynamics predicted by the maximum likelihood fit of the reproduction-selection model is in red. Right panel represents the relative fitnesses along with their corresponding support limits (≈ 95% confidence intervals) of the different genotypes (R_1_R_1_ was used as the reference, hence fixed to 1), estimated through a maximum likelihood approach for the parameters’ values in the reproduction-selection model (see methods)

After 11 generations of direct competition, the frequency of the R_1_ allele rose significantly, from 0.5 to ~ 0.63 in all three replicates, out-competing the R_2_^3^ duplicated allele (binomial test, all *p* < 0.004, Fig. 3A). To quantitatively estimate the fitness of the different genotypes, we then adjusted a model of reproduction-selection to the temporal genotypic data: we found that the heterozygous genotype (R_1_/R_2_^3^) conferred the highest fitness (w_R1R2_ = 1.23 [1.14–1.21] support limits) and that the R_1_/R_1_ genotype (w_R1R1_ = 1) had much higher fitness than R_2_^3^/R_2_^3^ (0.62 [0.56–0.68], Fig. 3 and Additional file 3: Fig. S1).

The strong fitness reduction incurred by resistant mosquitoes is thought to be associated with multiple pleiotropic deleterious effects, affecting many different life history traits, because of the reduced activity of the AChE1R [4, 7, 12, 28, 29, 42]. Accordingly, D alleles are thought to be selected because they reduce these deleterious effects by pairing a resistance copy (R, low AChE1 activity) and a susceptible copy (S, high AChE1 activity) in a heterogeneous duplication, thereby partly restoring the AChE1 activity to levels closer to those of susceptible alleles [21, 22]. However, the case of the homogeneous duplication R^x^ is less clear: we show in the present study that despite a higher global activity for R_2_^3^ compared to R_1_, the former allele induces higher selective disadvantages (Fig. 3), which is also what was observed for R^5^ *vs* R^3^ alleles in *An. gambiae* [22].

The less-than-strictly-additive AChE1R activity for the R_2_^3^ allele suggests that some deleterious effects could be associated with specific resistance alleles, potentially resulting from background deleterious mutations in the gene or its vicinity in the haplotype where the resistance mutation occurred. The fact that R_2_^3^/R_1_ heterozygotes appear to incur a higher fitness than both homozygotes supports this hypothesis (Fig. 3): if both alleles are weighted by linked deleterious mutations, they can complement each other, i.e. if they are different between the two alleles, the heterozygote would incur a higher fitness (a similar explanation has been proposed for the complementation of strongly deleterious D alleles in *C. pipiens* s.l. [17, 23]*.*). However, the overall activity remains higher for this allele compared to R_1_ (Fig. 1), and in *An. gambiae* it is the same *ace-1* sequence that is present in five or three copies [22]. It thus suggests that the architecture itself, i.e. the mere fact of carrying more copies, induces selective disadvantages. This structural “cost” could result from deleterious mutations trapped into the amplicons, from the breakpoints of the duplication being located in functional regions or from dosage imbalance for other genes embedded in the duplicated alleles that might disrupt biochemical equilibrium, as previously proposed [22, 23, 35]. Though none of these hypotheses are exclusive, the latter has been favored in the case of *An. gambiae* duplications: in this species, a ~ 200-kb amplicon encompassing 11 genes in addition to *ace-1* has been described, and a variant with a deletion of these other genes appears to be favored by selection in natural populations, probably because the deletion restores the gene balances [18]. Note that deleterious mutations in these closely linked genes could also explain the higher fitness of R_2_^3^/R_1_ heterozygotes (Fig. 3). There is thus a strong incentive to characterize the genomic structure of the *ace-1* duplications (either heterogeneous or homogeneous) in *C. pipiens* species complex too.

To summarize, the two *ace-1* R alleles present different evolutionary trade-offs: while having a higher copy number of resistance allele confers a higher resistance level, and thus higher selective advantage in presence of OP and CX insecticides, it is also associated with higher selective disadvantages, revealed in absence of insecticide. Although the mutations occurred independently in the different species, the same relationships among R copy number, resistance level and selective disadvantages have been described in *An. gambiae* [22]. Similarly, the R_2_^3^ duplicated allele would likely tend to be selected for in areas of intense selective pressure, its higher resistance surpassing its higher disadvantages, while the R_1_ single-copy allele would be favored in areas with more moderate intensity of treatment. This can reflect the ecology of the mosquito populations where these alleles were found: in the tropical areas where R_2_^3^ was found, *C. quinquefasciatus* is the year-long vector of several viruses and thus probably subjected to more intense and regular treatments than in the Mediterranean area where R_1_ is found and where *C. pipiens* s.s. is less a vector than a summer nuisance (the female diapauses in winter). The genotyping of natural populations to look specifically for the presence of R^x^ homogeneous duplications of the *ace-1* locus could confirm this hypothesis. It would also allow us to understand whether the number of copies is as variable as in *An. gambiae* (at least up to 6 copies [22]), whether the R^x^ alleles are only found in *C. quinquefasciatus* or are also found in *C. pipiens* s.s. and, if so, if they are found in populations experiencing higher treatment intensities. Finally, the recurrent selection of homogeneous duplications of the *ace-1* resistance copies in phylogenetically distant species complexes (e.g. in *Anopheles* [18, 22, 30] and in *Culex*, this study), along with the high diversity of heterogeneous duplications already described in both species [7, 17, 23, 30, 35, 43–45], provides further support for a very high duplication rate of the *ace-1* loci. It also highlights the versatility of adaptive responses that can result from such structural variants (i.e. as opposed to simple SNP): from a more quantitative resistance advantage resulting, at least in part, from the increased amount of protein produced for the homogeneous duplications (as also seen for metabolic resistances like esterases or P450 monooxygenases [46]) to a more qualitative advantage for the heterogeneous duplications that allow the fixation of a heterozygote advantage selected in more variable environments [5, 23]. Note however that the recurrent selection of architectures such as homogeneous duplications in distant lineages calls for more functional research to understand how producing more AChE1R proteins leads to higher resistance levels.

## Conclusion

In *C. pipiens* species complex many different genetic architectures encompassing the *ace-1* locus exist for resistance to OPs and CXs insecticides, which are each associated with a different evolutionary trade-off: the single-copy resistance allele provides resistance but is associated with a high selective disadvantage in absence of insecticides, while homogeneous duplications provide even higher resistance levels but are associated with higher selective disadvantages. Not only different genetic architectures could represent various steps along an adaptive walk, but there also are many ways to answer to the various intensities of selective pressure. While inspiring from an evolutionary perspective, the vector management view is clearly worrying, as this ‘toolbox’ allows mosquito populations to finely and quickly adjust to local treatment strategies in natural populations (particularly if one considers the various heterozygous combinations between the different alleles), which definitely represents a hindrance to vector control policies.

## Supplementary Information

**Additional file 1: **Sequence alignment for R1 (SR) and R2 (SRQ). The primers used for the PCR amplification are indicated (light gray) as well as the restriction sites for the *BfaI *and *AluI *enzymes (darker gray, the triangles indicate restriction cuts). Mutations between the two sequences are in bold.

**Additional file 2**. This file basically contains all the raw data supporting each analysis.

**Additional file 3: Table S1**. Analysis of variance of model 1. **Table S2.** Bioassay analyses. **Figure S1.** Reproduction-selection model likelihood profiles.

## Data Availability

All data generated or analyzed during this study are included in this published article and its supplementary information files: “Additional file 2.”
