# Supplementary material for: Evolutionary trade-offs associated with copy number variations in resistance alleles in Culex pipiens mosquitoes
Source: Parasit Vectors. 2022 Dec 22;15:484. doi: 10.1186/s13071-022-05599-8 (PMC9783466; doi:10.1186/s13071-022-05599-8)
Supplement: Supplementary file 1 — Additional file 1: Sequence alignment for R1 (SR) and R2 (SRQ). The primers used for the PCR amplification are indicated (light gray) as well as the restriction sites for the BfaI and AluI enzymes (darker gray, the triangles indicate restriction cuts). Mutations between the two sequences are in bold. [file 13071_2022_5599_MOESM1_ESM.pdf]

Additional File 1 : Sequence alignment for R_1 (SR) and R_2 (SRQ). The primers used for the PCR amplification are indicated (light gray), as well as the restriction sites for the *BfaI* and *AluI* enzymes (darker gray, the triangles indicate where restriction cuts). Mutations between the two sequences are bolded.

```
         1                                                 50
R1-SR    GCGCGAGCAT ATCCATAGCA CTACGACCCG GCGGCGTGGC CTGACCCGGA
R2-SRQ   GCGCGAGCAT ATCCATAGCA CTACGACCCG GCGGCGTGGC CTAACCCGGA
         Primer Intron2dir1
         51                                               100
R1-SR    GGGAGTCCAG CTCCGGTGAG TTCTGGGAGG GG........ GCCATTT.CC
R2-SRQ   GGGAGTCCAG CTCCGGTGAG TTCTAGGAGG GGCTTTTGGG GTCATTTGCC
                                 BfaI
         101                                              150
R1-SR    CTTTGCTAAC TAATTTACTC CAATTTTCGT AGATGCCACC GACTCGGACC
R2-SRQ   CTTTGCTAAC TAATTCACTC CAATTTTCGT AGATGCCACC GACTCGGACC

         151                                              200
R1-SR    CACTGGTAAT AACGACGGAC AAGGGCAAAA TCCGTGGAAC GACACTGGAA
R2-SRQ   CACTGGTCAT AACGACGGAC AAAGGCAAAA TCCGTGGAAC GACACTGGAA

         201                                              250
R1-SR    GCGCCAAGTG GAAAGAAGGT GGACGCATGG ATGGGCATTC CGTACGCGCA
R2-SRQ   GCGCCTAGCG GAAAGAAGGT GGACGCATGG ATGGGCATTC CGTACGCGCA
             BfaI
         251                                              300
R1-SR    GCCCCCGCTG GGTCCGCTCC GGTTTCGACA TCCGCGACCC GCCGAAAGAT
R2-SRQ   GCCTCCGCTG GGTCCGCTCC GGTTTCGACA TCCGCGACCC GCCGAAAGAT

         301                                              350
R1-SR    GGACCGGTGT GCTGAACGCG ACCAAACCAC CCAACTCCTG CGTCCAGATC
R2-SRQ   GGACCGGTGT GCTGAACGCG ACCAAACCGC CCAACTCCTG CGTCCAGATC

         351                                              400
R1-SR    GTGGACACCG TGTTCGGTGA CTTCCCGGGG GCCACCATGT GGAACCCGAA
R2-SRQ   GTGGACACCG TGTTCGGTGA CTTCCCGGGG GCCACCATGT GGAACCCGAA

         401                                              450
R1-SR    CACACCCCTC TCGGAGGACT GTCTGTACAT CAACGTGGTC GTGCCAAGGC
R2-SRQ   CACACCGCTC TCGGAGGACT GTCTGTACAT CAACGTGGTC GTGCCACGGC

         451                                              500
R1-SR    CGAGGCCCAA GAATGCCGCT GTCATGCTGT GGATCTTTGG GGGTAGCTTC
R2-SRQ   CCAGGCCCAA GAATGCCGCC GTCATGCTGT GGATCTTCGG GGGTAGCTTC
                                                       AluI
         501                                              550
R1-SR    TACTCCGGGA CTGCCACGTT GGACGTGTAC GATCATCGGA CGCTGGCCTC
R2-SRQ   TACTCCGGGA CTGCCACGCT GGACGTGTAC GACCACCGGA CGCTGGCCTC

         551                                          597
R1-SR    GGAGGAGAAC GTGATCGTGG TTTCGCTGCA GTACCGTGTC GCAAGTC
R2-SRQ   GGAGGAGAAC GTGATCGTAG TTTCGCTGCA GTACCGTGTC GCAAGTC
                                    Primer CpEx3rev
```
