# Supplementary material for: Evolutionary trade-offs associated with copy number variations in resistance alleles in Culex pipiens mosquitoes
Source: Parasit Vectors. 2022 Dec 22;15:484. doi: 10.1186/s13071-022-05599-8 (PMC9783466; doi:10.1186/s13071-022-05599-8)
Supplement: Supplementary file 3 — Additional file 3: Table S1. Analysis of variance of model 1. Table S2. Bioassay analyses. Figure S1. Reproduction-selection model likelihood profiles. [file 13071_2022_5599_MOESM3_ESM.docx]

# Evolutionary trade-offs associated with copy number variations in resistance alleles in *Culex pipiens* mosquitoes.

Pascal Milesi, Jean-Loup Claret, Sandra Unal, Mylène Weill and Pierrick Labbé

**Additional information**

**Figure S1: Reproduction – selection model likelihood profiles.**

**Table S1: Analysis of variance of model 1.**

| **Effect** |  | **df** |  | **Sum. Sq** |  | **Mean Sq** |  | ***F*-value** |  | ***p*-value** |
| --- | --- | --- | --- | --- | --- | --- | --- | --- | --- | --- |
| Strain |  | 1 |  | 467.97 |  | 467.97 |  | 269 |  | < 2e-16 |
| Plate |  | 1 |  | 0 |  | 0 |  | 0.00 |  | 0.99 |
| Sex |  | 1 |  | 6.75 |  | 6.75 |  | 3.88 |  | 0.05 |
| Strain : Plate |  | 1 |  | 0.01 |  | 0.01 |  | 0.01 |  | 0.92 |
| Strain : Sex |  | 1 |  | 1.79 |  | 1.79 |  | 1.03 |  | 0.31 |
| Plate : Sex |  | 1 |  | 0.44 |  | 0.44 |  | 0.25 |  | 0.62 |
| Strain : Plate : Sex |  | 1 |  | 0.02 |  | 0.02 |  | 0.01 |  | 0.92 |
| Residuals |  | 84 |  | 146.39 |  | 1.74 |  |  |  |  |

**Table S2: Bioassay analyses.**

| **Strains** |  | ***Regression*** | |  | ***Heterogeneity*** | |  | ***Linearity test*** | | |  | **Lethal doses (95% CI)** | | |  | **Resistance Ratios (95% CI)** | | |
| --- | --- | --- | --- | --- | --- | --- | --- | --- | --- | --- | --- | --- | --- | --- | --- | --- | --- | --- |
|  |  | ***b*** | ***a*** |  | ***h*** | ***g*** |  | ***X^2^*** | ***df*** | ***p*** |  | **LD_25_** | **LD_50_** | **LD_95_** |  | **RR_25_** | **RR_50_** | **RR_95_** |
| SLAB |  | 4.2 ± 0.9 | 13 ± 2.7 |  | 3.9 | 0.22 |  | 8.7 | 9 | 0.46 |  | 4.10^-4^ (0 – 5.10^-3^) | 1.10^-3^ (0 – 0.07) | 2.10^-3^ (0 – 0.13) |  | 1 (0.8 – 1.26) | 1 (0.8 – 1.2) | 1 (0.7 – 1.5) |
| SR |  | 3.8 ± 0.8 | 10 ± 2.1 |  | 5.4 | 0.32 |  | 11 | 6 | 0.08 |  | 2.10^-3^ (0 – 0.14) | 3.10^-3^ (0 – 0.19) | 8.10^-3^ (0 – 0.36) |  | 4.4 (3.4 – 5.8) | 4.6 (3.7 – 5.7) | 5.1 (3.2 – 8.1) |
| SRQ |  | 2.6 ± 0.3 | 5 ± 0.7 |  | 2.5 | 0.08 |  | 9.3 | 11 | 0.59 |  | 5.10^-3^ (1.10^-4^ – 0.05) | 9.10^-3^ (2.10^-4^ – 0.07) | 4.10^-2^ (2.10^-3^ – 0.23) |  | 12 (9.3 - 15) | 15 (12 – 18) | 26 (15 – 47) |

*b:* slope

*a: intercept*
